# Deep brain stimulation-induced normalization of hippocampal synchrony in a transgenic rat model of Alzheimer's disease

**DOI:** 10.7150/thno.110292

**Published:** 2025-09-22

**Authors:** Andrea Trevisiol, Tina Beckett, Monica Bell Vila, Mary Hill, Joanne McLaurin, Bojana Stefanovic

**Affiliations:** 1 Physical Sciences, Sunnybrook Research Institute, Toronto, ON, Canada.; 2 Biological Sciences, Sunnybrook Research Institute, Toronto, ON, Canada.; 3 Department of Laboratory Medicine and Pathobiology, Temerty Faculty of Medicine, University of Toronto, Toronto, ON, Canada.; 4 Department of Medical Biophysics, University of Toronto, Toronto, ON, Canada.

**Keywords:** Alzheimer's disease, TgF344-AD rats, neuromodulation, deep brain stimulation, electrophysiological recordings, local field potentials

## Abstract

**Background and Aim:** Alzheimer's disease (AD) is a progressive neurodegenerative disorder characterized by disrupted neural network dynamics and neuronal loss. Deep brain stimulation (DBS) may restore network function and abate cognitive deficits. In a transgenic rat model of AD, we investigated the dependence of hippocampal neuronal activity on a range of DBS parameters, aiming to identify stimulation conditions that transiently restore impaired network function.

**Material and Methods:** We used 16-month-old TgF344-AD and NTg rats under light anesthesia and performed simultaneous DBS and high-resolution intracerebral recordings in the hippocampus using a linear multielectrode array. DBS was delivered in bipolar mode, at varying frequencies, amplitudes and duration, while monitoring local field potentials (LFP) and spiking activity. Phase-amplitude coupling (PAC), neuronal power, and firing rates were analyzed prior to and following DBS. Linear mixed effects models were used to evaluate the influence of genotype, sex, and stimulation parameters on the electrophysiological markers.

**Results:** With increasing DBS frequency and amplitude, hippocampal power and PAC rose in all rats, particularly within the delta-theta range. When compared to NTgs, TgAD rats showed attenuated power but increased PAC responses to DBS. Low frequency DBS induced higher entrainment in the post- relative to during-DBS period in all animals. Compared to their non-transgenic littermates, TgAD rats showed reduced entrainment responses.

**Conclusions:** These findings demonstrate that hippocampal responses to DBS have a parameter-dependent profile that is differentially modulated by AD pathology. Our study provides a foundation for tailoring DBS parameters to compensate for distinct neuronal deficits in established AD, supporting the use of electrophysiological biomarkers to guide individualized neuromodulation strategies

## Introduction

Alzheimer's disease (AD) is a progressive neurodegenerative disease characterized by the accumulation of amyloid-beta (Aβ) plaques and hyperphosphorylated tau tangles 1. AD patients show progressive memory and cognitive impairments linked to alterations in neural network dynamics, synchronization, and connectivity (for an in-depth review see 2). Notably, network changes have received considerably less research attention than pathological and genomic AD biomarkers 3. One prominent AD network alteration is reduced cross-frequency coupling between prefrontal gamma amplitude and hippocampal theta phase 4,5. In addition to theta-gamma coupling deficits, hippocampal network dysfunction in AD includes impaired sharp-wave ripples and disrupted hippocampal-entorhinal coordination, preceding cognitive decline 6. Altered inhibitory circuitry, particularly GABAergic synapse loss, disrupts the excitation-inhibition balance, leading to hyperexcitability and network asynchrony 7,8. These disturbances extend across frequency bands, reducing power, coherence, and prefrontal-hippocampal connectivity 9. Moreover, tau pathology, independent of amyloid, impairs CA1 complex-spike bursting and associated rhythms essential for memory, underscoring the need for interventions that restore circuit synchrony 10. Many of these network abnormalities likely stem from impaired neurotransmission and post-synaptic dysfunction due to reduced presenilin function, which disrupts synaptic calcium homeostasis 11, and leads to Aβ accumulation 12. The marked electrophysiological alterations in the pre-symptomatic phase of AD have prompted efforts to systematically characterize their temporal dynamics, both as potential early biomarkers and as quantitative indicators of disease progression 2. Current therapeutic treatments - both symptomatic and disease-modifying - have been largely ineffective in improving AD patients' cognition and overall quality of life 13. In an effort to improve cognitive symptoms, few pioneering studies have tested the effectiveness of electrical stimulation through deep brain stimulation (DBS) for managing AD 14. Since its introduction as a neurotherapeutic in Parkinson's disease (PD) patients who did not adequately respond to levodopa 15, DBS has shown promising results for attenuation of clinical symptoms of severe depression, epilepsy, and AD 16,17. While the role of DBS in inhibiting the hyperactive subthalamic nucleus in PD is well-established, the targets and criteria for DBS in AD and other neurodegenerative diseases remain ill-defined.

Hitherto studies investigating DBS for memory enhancement 18 and stabilization of AD cognitive decline have reported diverse outcomes 19,20, and the cellular changes underpinning these outcomes remain poorly understood 19. The efficacy of DBS in modulating and potentially restoring brain function seems to be contingent on brain disease stage and anatomical targeting of relevant brain areas, as well as the DBS amplitude, frequency, stimulation duration, and duty cycle 21. The preponderant DBS targets have been areas directly connected to the hippocampus (HIP): the fornix, an efferent white matter tract emerging from the HIP 22 and the nucleus basalis of Meynert that projects to the neocortex, amygdala, and thalamus and is involved in memory formation 23. Inhibition of these targets using high-frequency stimulation has led to mixed outcomes, as indicated by a recent meta-analysis 20 and remains confounded by our limited understanding of their functional connections with the HIP. Neuromodulating regions central to AD pathology —such as the HIP, entorhinal cortex 24, or prefrontal cortex— are thus anticipated to elicit greater clinical benefits, but challenging to achieve via non-invasive neuromodulation (gamma-frequency sensory stimulation and temporal interference 25,26) due to the limited spatial and temporal precision of non-invasive approaches. To advance neuromodulatory strategies, more reliable biomarkers are needed to guide intervention efficacy at every stage of the disease. In addition to molecular biomarkers 14, a promising yet understudied biomarker is neuronal network activity, which can be monitored in real time, using the same electrode through which DBS can be delivered, so as to enable high-precision, closed-loop stimulation. We presently examined the effects of hippocampal DBS by high-sensitivity monitoring of neuronal network activity via intracerebral electrophysiological recordings. We acutely and directly targeted the hippocampal circuit in 16-month-old TgF344-AD rats, which display advanced amyloid and tau pathologies in combination with frank neuronal loss and pronounced impairments in memory, executive function, and long-term extinction memory 27,28 recapitulating the advanced stage of AD, when invasive interventions are most likely to be applied clinically. Through the same linear multi-array electrode, we monitored the effects on network activity and neuronal spiking. By modeling hippocampal network responses to DBS across a range of stimulation parameters, we established a predictive framework to inform DBS parameter selection and support its therapeutic application to counteract AD-induced hippocampal dysfunction.

## Materials and Methods

We used TgF344-AD rats as an Alzheimer's disease model 29, following approved ethical guidelines set by the Animal Care Committee of the Sunnybrook Research Institute. Sex-balanced, age-matched TgAD and NTg rats were kept under a 12-hour light-dark cycle, with physiological monitoring during anesthesia.

Isoflurane anesthesia (2-2.5%) enabled the placement of a 3 mm craniotomy at AP -3.5 mm, ML -2.5 mm. After electrode positioning, rats were transitioned to intravenous propofol anesthesia (40-50 mg/kg/hr) via tail vein catheter for stable electrophysiological recordings. A double-shank 2x6 linear multielectrode array (LMA) was inserted into the left hippocampus (HIP), at a depth of -6.0 mm from the surface, ensuring full dorsoventral HIP coverage. Signals were recorded under low isoflurane (1.2-1.5%) and propofol at 24.4 kHz and divided into low-frequency (LFP: 0.3-300 Hz) and high-frequency (AP band: 0.75-3 kHz) components for analysis. Modulation index (MI), representing phase-amplitude coupling (PAC) and power, was derived from the LFP. Firing rate (FR) and phase-locking consistency (PPC) were computed from AP band activity (spiking events), and the number of responding units (NRU) was determined based on FR and PPC changes relative to a shuffled null distribution. DBS was delivered in bipolar mode via the most dorsal and ventral contacts of the LMA using 0.05 ms square pulses at amplitudes of 2, 5, 10, and 20 µA (corresponding to 20, 50, 100, and 200 μC/cm² per pulse) and frequencies of 1 Hz,10, 20, 40, 100, and 130 Hz. Each DBS block lasted 10 seconds, followed by a 20-second recovery. Linear mixed-effects (LME) models were employed to examine the effects of genotype, sex, DBS amplitude/frequency, and regional variability on electrophysiological parameters, with model selection guided by Akaike Information Criterion. Pairwise comparisons were conducted following ANOVA with Tukey-Kramer correction.

Further details on the experimental procedures and analyses are provided in the supplementary materials.

### Data availability

Raw and processed data acquired in these experiments, along with the analysis scripts and models, are available upon request.

## Results

**Figure 1:**
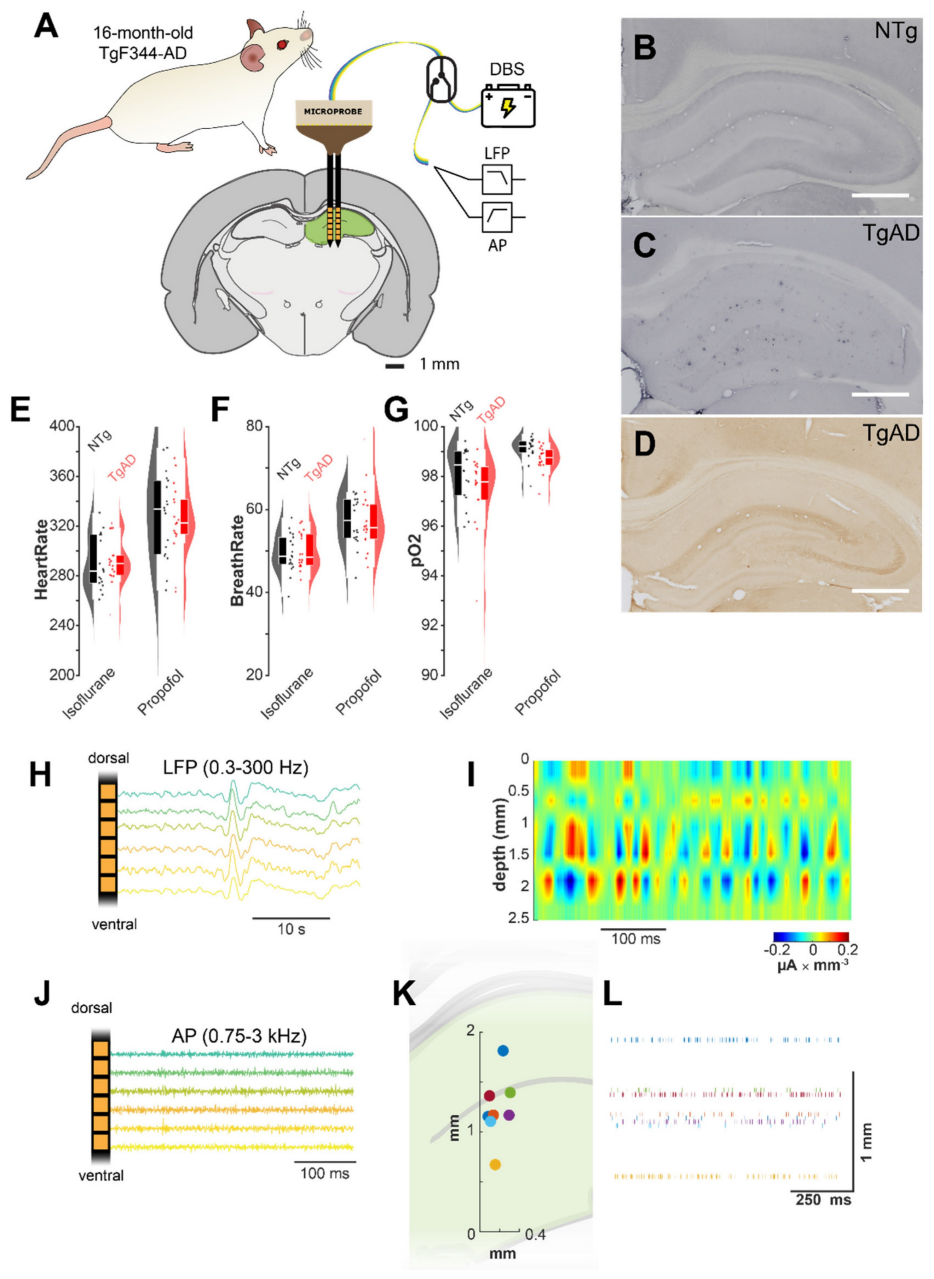
DBS e*xperimental setup.* (A) The dual-shank linear multi-channel array electrodes used for DBS and LFP recording were lowered along the dorsoventral axis into the hippocampus of 16-month-old TgAD rats or their NTg littermates; a switching head stage allowed for fast switching between recording and stimulation. (B-C) Widespread amyloid plaque deposition in the hippocampus of 16 m.o. TgAD rats (C) vs. NTg (B) as shown by 6F3D Aβ42-antibody. (D) Hippocampal distribution of hyperphosphorylated tau in 16 m.o. TgAD rats detected by PHF1-antibody. (E-G) Physiological parameters (E: heart rate, F: breathing rate, G: arterial O_2_ saturation) of TgAD and NTg rats undergoing the transition from isoflurane to continuous infusion of low-dose propofol anesthesia. (H) Representative extracellular recordings in the 0.3-300Hz band (local field potentials, LFP). (I) Representative current source density (CSD) analysis of hippocampal network activity in TgAD rats localizes current sinks (red, positive voltage deflections) and sources (blue, negative voltage deflection). (J) Representative extracellular recordings in the high frequency band (action potentials, AP_band_): 0.75-3kHz. (K) Representative spiking unit distribution in the hippocampus along the x-z axis, identified by the clustering analysis. (L) Representative raster plot of the spiking units in a sample animal. Scale bar in B-D: 1 mm.

To evaluate the acute effects of DBS on hippocampal (HIP) networks in advanced AD, we used a double-shank linear electrode array targeting the HIP of 16-month-old TgF344-AD rats (Figure 1A). The HIP of these animals showed a significant burden of Aβ and phosphorylated tau (Figure 1B-D). A fast-switching headstage enabled alternating between DBS stimulation and recordings of low-frequency (LFP) and high-frequency (AP band) extracellular activity (Figures 1B, 1C).

### Effects of DBS on LFP power

**Figure 2:**
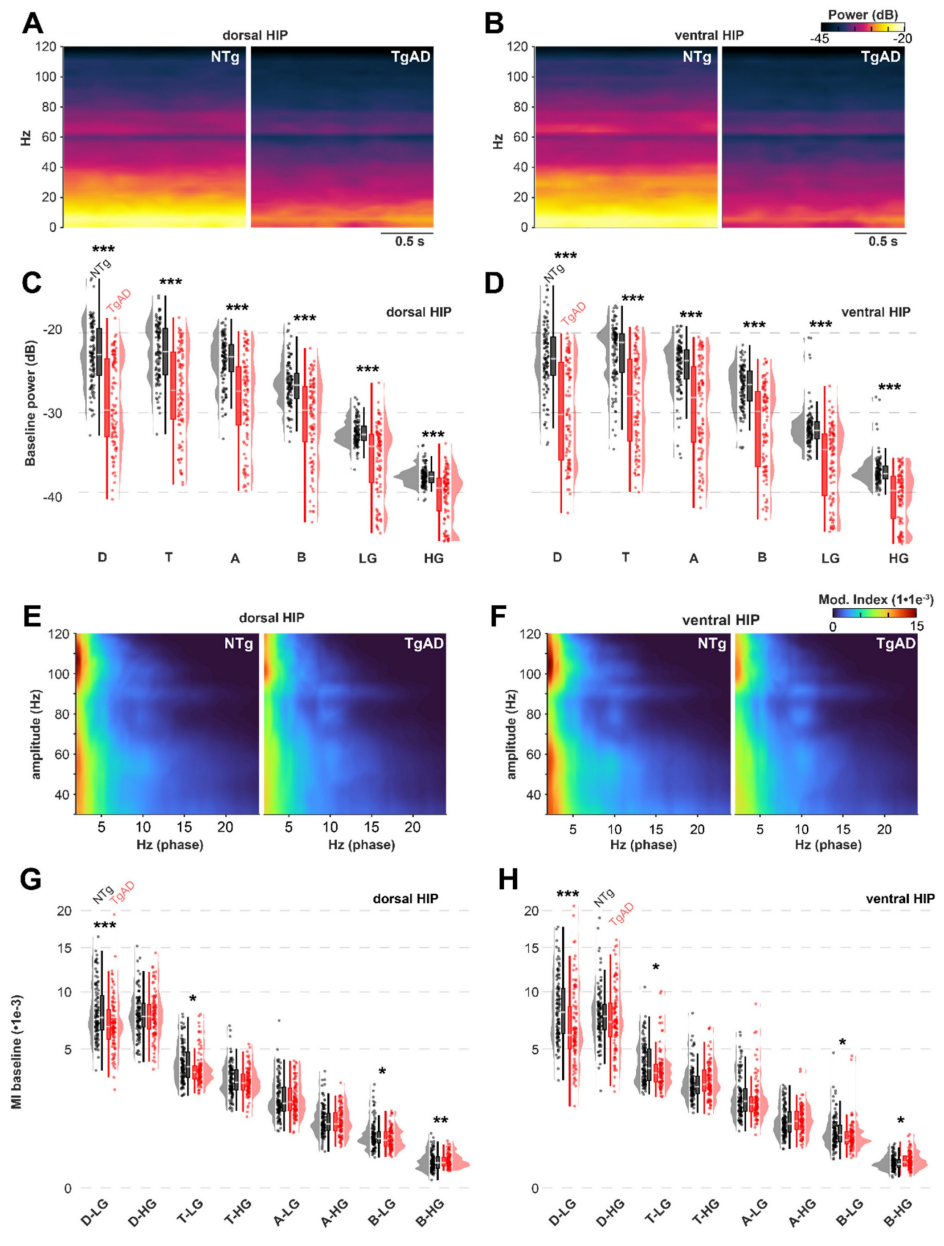
*Attenuation of TgAD hippocampal resting power and synchronization under propofol anesthesia at baseline (no stimulation).* (A-B) LFP power spectrum analysis in the dorsal (A) and ventral (B) hippocampus indicates a decrease in power in the TgAD cohort (right image) across the frequency spectrum (0.1-120 Hz) compared to that of the NTg (left) cohort. (C-D) LFP power quantification in dorsal (C) and ventral (D) hippocampus in different bands (delta D: 0.1-4Hz; theta T: 4-8Hz; alpha A: 8-12Hz; beta B: 12-30 Hz; low-gamma LG: 30-60Hz and high-gamma HG: 60-120Hz) showing attenuation in TgAD (red) rats across all bands. (E-F) Phase-amplitude coupling analysis of the LFP signal rendered as comodulograms in dorsal (E) and ventral (F) hippocampus. (G-H) Modulation index (MI) across all combinations of the low-frequency band (D, T, A, B) phases and high-frequency band (LG/HG) amplitudes in TgAD (red) and NTg (black) rats. Images in A, B, E, and F display averages across all subjects. *Statistical significance between genotypes is indicated with asterisks:* * p < 0.05, ** p < 0.01, *** p < 0.001, based on Tukey-adjusted post-hoc comparisons. Tg n=17, NTg n=19.

**Figure 3:**
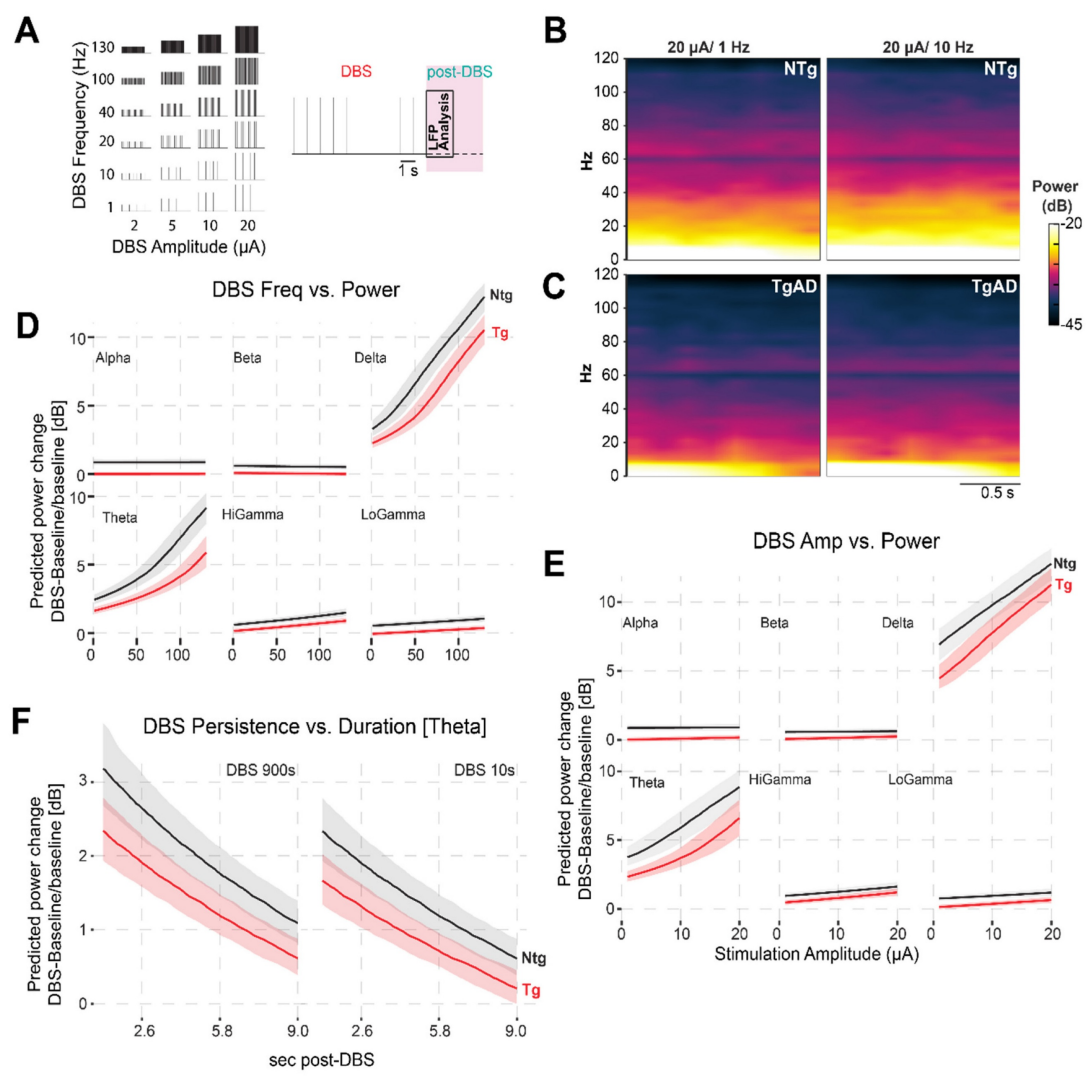
*Attenuation of TgAD DBS-induced hippocampal power changes depends on frequency and amplitude.* (A) combination of DBS parameters (frequencies and amplitudes) used (left) and schematic of the stimulation protocol (right). Deep brain stimulation (DBS) was delivered to the hippocampus in 10-second trains at all combinations of frequencies (1-130 Hz) and amplitudes (2-20 μA), followed by a post-stimulation period used for LFP analysis. (B-C) Time-frequency representations of hippocampal power in NTg (B) and TgAD (C) rats following DBS at 20 μA for 1 Hz (left) and 10 Hz (right). Compared to NTg animals, TgAD rats show a reduced power increase in the low-frequency range following DBS. (D) Frequency-dependent effects of DBS on power across canonical oscillation bands (delta, theta, alpha, beta, Low gamma, High gamma), plotted separately for NTg (black) and TgAD (red) rats. Linear mixed-effects model predictions show a strong positive relationship between DBS frequency and post-stimulation power, particularly in low-frequency bands, with attenuated responses in TgAD rats. (E) Same analysis as in panel (D), but for stimulation amplitude (2-20 μA). Increased amplitude leads to enhanced power responses, again less prominent in TgAD animals. (F) Analysis of post-stimulation persistence of theta power changes following either short (10s) or long (900s) DBS trains. DBS-induced increases in theta power decay similarly over time in both genotypes, with NTg rats consistently showing larger power changes.

**Figure 4:**
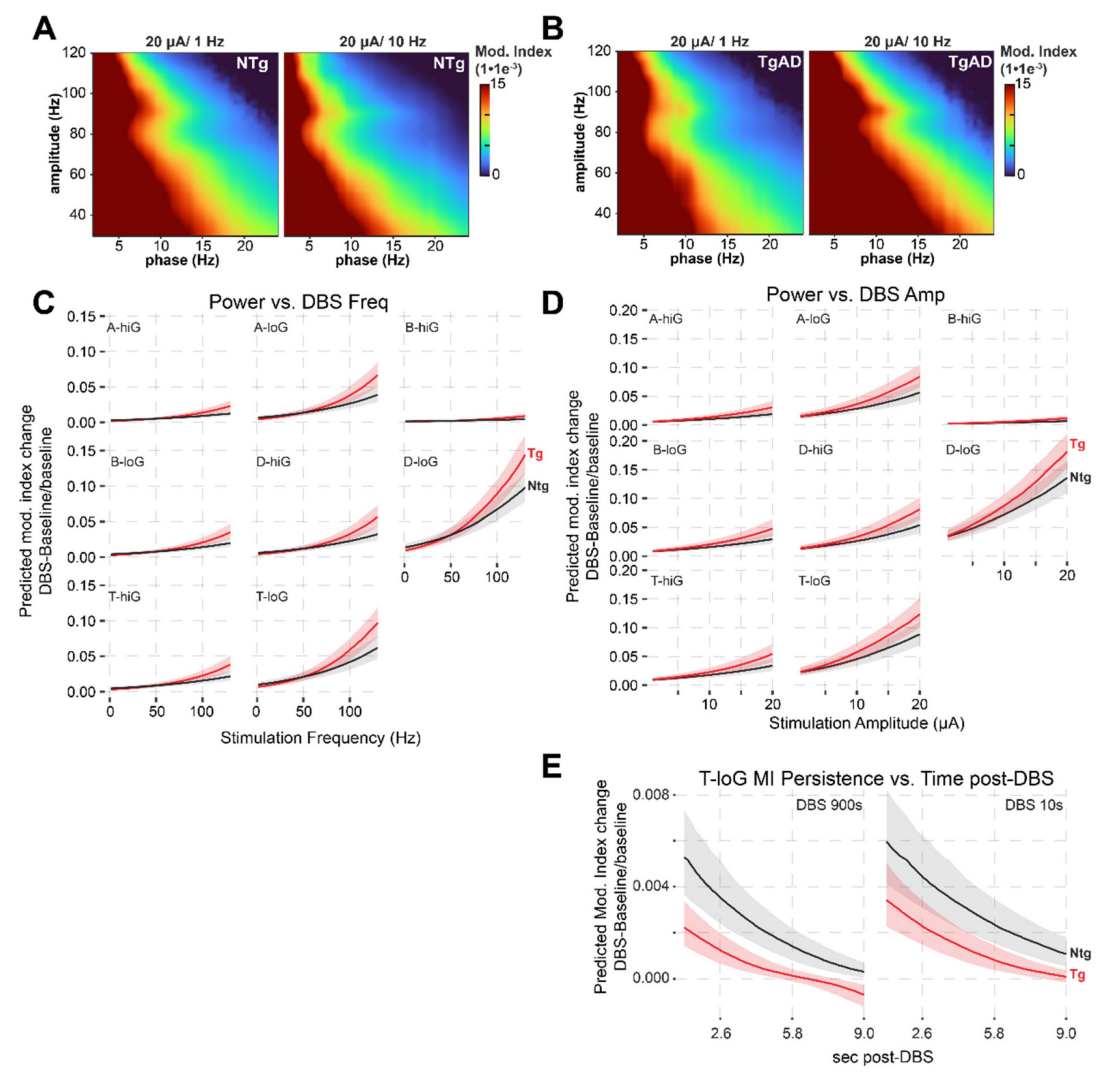
*DBS-induced increases in PAC (modulation index) in the hippocampus of TgAD rats, in frequency- and amplitude-dependent manner.* (A) The averaged modulation index (MI) map of LFP following DBS at 20 µA/ 1 Hz (left) and 20 µA/ 10 Hz (right) in NTg rats. (B) The corresponding MI co-modulation map in TgAD rats. (C) Frequency-dependent effects of DBS on PAC across low-high frequency combinations of oscillation band (delta (D), theta (T), alpha (A), beta (B) vs. Low (loG) and High (hiG) gamma), plotted separately for NTg (black) and TgAD (red) rats. Linear mixed-effects model predictions showed a positive relationship between DBS frequency and post-DBS modulation, particularly in low-frequency bands, with attenuated responses in TgAD rats at low DBS frequencies and increased at high DBS frequencies. (D) Same analysis as in panel (C), but for stimulation amplitude (2-20 μA). Increased amplitude leads to enhanced modulation index responses, more prominently in TgAD animals at higher amplitudes. (E) Analysis of post-stimulation PAC response persistence of MI changes in theta-low gamma modulation following either short (10 s) or long (900 s) DBS trains. DBS-induced increases in theta-low gamma modulation decay at similar rates over time in both genotypes, with NTg rats showing larger changes in modulation overall.

At baseline, TgAD rats displayed significant HIP network dysfunction, manifested as reduced power across all frequency bands (Figure 2C-D): these effects included genotype-sex interaction (Figure S1). For instance, dorsal HIP theta power was reduced in TgAD animals (-5.0 dB; 95% CI: -5.6 to -4.6) relative to NTgs. To test whether DBS could ameliorate these impairments, we systematically varied stimulation parameters — frequency (1-130 Hz), amplitude (1-20 µA), and duration (10 vs. 900 s) — and quantified the relative change in LFP power from baseline (DBS-baseline, ΔPower; Figures 3-4). To describe the power responses to DBS in TgAD and NTg hippocampi, we modeled the change in power (ΔPower) as a function of stimulation frequency (1-130 Hz), amplitude (1-20 μA) and as a function of time following DBS offset (post-DBS window, 0-10 sec) using linear models. Both DBS amplitude and frequency increased LFP power independently (p=0.013 and p=0.007 respectively), with a significant interaction effect between the two (p <0.001). Notably, power enhancement with increasing DBS intensity was higher in the lower-frequency oscillations (delta-theta, Figure 3D-E). Stimulation duration also modulated the persistence of power changes (p = 0.01; Figure 3F). DBS amplitude, but not frequency, had significant genotype-specific effects on hippocampal LFP power (p < 0.001), indicating that genotype (i.e. AD pathology) is more sensitive, in terms of power changes to DBS, to amplitude selection. While DBS produced increases in neuronal power, the magnitude of this increase was higher in NTg than in Tg animals, across all oscillation bands (Figure 3D-F and Figure S2B-D), irrespective of other DBS parameters. For example, 40Hz/9μA DBS induced +3.2 dB (95% CI: 2.7-3.8 dB) increase in NTg vs +2.0 dB (95% CI: 1.7 to 2.4 dB) in TgAD in the theta band of the dorsal HIP, i.e. DBS responses in power in the HIP of TgAD rats were attenuated when compared to those in the HIP of NTgs.

Given these differences, we used our fitted model to identify DBS parameter combinations that could compensate for HIP neuronal deficits in TgAD animals. For instance, based on the observed -5.0 dB resting power deficit in theta band of the dorsal HIP, the model predicted that a 10-second DBS pulse at 19.0 µA and 34.9 Hz would transiently restore LFP power to levels comparable to NTgs. Similarly, post-stimulation analysis (Figure 3F) showed that in the AD cohort, dorsal HIP theta power reached 50% of its DBS-induced maximum approximately 4s and 3s after the end of stimulation for 900 s and 10 s DBS durations, respectively, exemplifying the potential of our parameter-response model to guide the optimization of DBS protocols based on disease-specific network impairments.

### DBS effects on phase amplitude coupling

Consistent with the advanced stage of the AD pathology and the widespread reduction in hippocampal LFP power, TgAD rats also exhibited impaired network coordination at baseline, as reflected by a reduction in phase-amplitude coupling (PAC; Figure 2E-H). Specifically, the modulation index (MI) was significantly decreased in several PAC frequency pairings, including delta-Low gamma (D-LG), theta-Low gamma (T-LG), and beta-Low gamma (B-LG). For example, in the D-LG combination, baseline MI in NTg rats was 0.0084 (95% CI: 0.0080 to 0.0086), while TgAD rats showed a significantly lower MI of 0.0072 (95% CI: 0.0070-0.0079; p < 0.001; Figure 2E-G).

To assess whether DBS could mitigate synchronization changes in the HIP of TgAD, we quantified DBS effects on network coordination by reporting changes in MI (ΔMI) from baseline to DBS across PAC pairings (Figure 4A-E). These changes were modeled using a linear mixed-effects model that included fixed effects for genotype, sex, DBS parameters (amplitude and frequency), and their interactions (Figure 4C-E, Figure S3A-H). The model revealed a significant genotype:sex interaction on ΔMI (p = 0.037), and both DBS amplitude and frequency significantly influenced MI changes individually (p < 0.001), and through their interaction (p < 0.001). Similarly to LFP power changes, increases in DBS amplitude and frequency were correlated with increases in MI. For instance, in the D-LG PAC of the dorsal HIP of NTg rats, DBS at 40Hz/9μA increased MI by +0.0267 (95% CI: 0.0200 to 0.0355) while the increase was +0.0251 (95%CI: 0.0188 to 0.0334) in TgAD. These correspond to relative increases of +318% and +349%, respectively, indicating that DBS induced a marked enhancement of network coordination that exceeded even NTg baseline levels. Importantly, all tested DBS parameter combinations produced changes in MI that exceeded the minimum required to normalize PAC in TgAD rats. For example, in the dorsal HIP and for D-LG coupling, a ΔMI of 0.0012 would be required to restore PAC to NTg baseline levels, yet even the smallest DBS-induced change observed in TgAD rats was 0.0015. This indicates that DBS can elicit at least transient hypersynchronization.

### Comparison of DBS effects on power vs. modulation index

Interestingly, network power was influenced by DBS amplitude alone, in a genotype-dependent manner (p < 0.001). LFP power consistently increased less in TgAD than in NTg rats across all DBS parameters (Figure 3D-F). Of note, power levels recorded in both cohorts were affected by anesthesia. To minimize anesthetic-specific confounding effects, we compared DBS responses obtained under propofol with DBS responses under isoflurane, which shows a less specific effect on GABA-A than does propofol 30,31. Notably, TgAD animals showed larger theta power increases under isoflurane than they did under propofol whereas NTg animals displayed relatively consistent power responses to increasing DBS frequency and amplitude under both anesthetics (Figure S2E-H).

In contrast to power, MI responses were influenced by both DBS frequency and amplitude in a genotype-dependent manner (both p < 0.001), indicating a significant interaction between stimulation parameters and genotype, and suggesting that HIP network synchronization is more sensitive to DBS frequency. The TgAD MI increased with increasing stimulation amplitude and frequency, and this TgAD MI response exceeded that of the NTg cohort (Figure 4C-D). Indeed, at higher stimulation levels (DBS >50Hz and >5 μA) TgAD MI responses to DBS surpassed those of NTg rats (Figure 4C-D). This increase in PAC observed in the HIP network of TgAD was very large at higher frequencies (e.g. at 130Hz for theta-low-gamma, MI reached a 28-fold increase compared to baseline values, Figure 2G-H and Figure 4C-D): the emergent DBS-induced hypersynchrony may have resulted from an AD-induced alteration in inhibitory control mechanisms. Taken together, these findings suggest that hippocampal circuits in TgAD rats exhibit not only blunted power responses to DBS but also very large increases in PAC in response to DBS, potentially *indicative of pathological hypersynchrony under high-frequency or high-amplitude DBS conditions*. PAC responses were largely consistent across genotypes among the anesthetics tested (Figure S3E-H).

### Spiking activity responses to DBS

**Figure 5:**
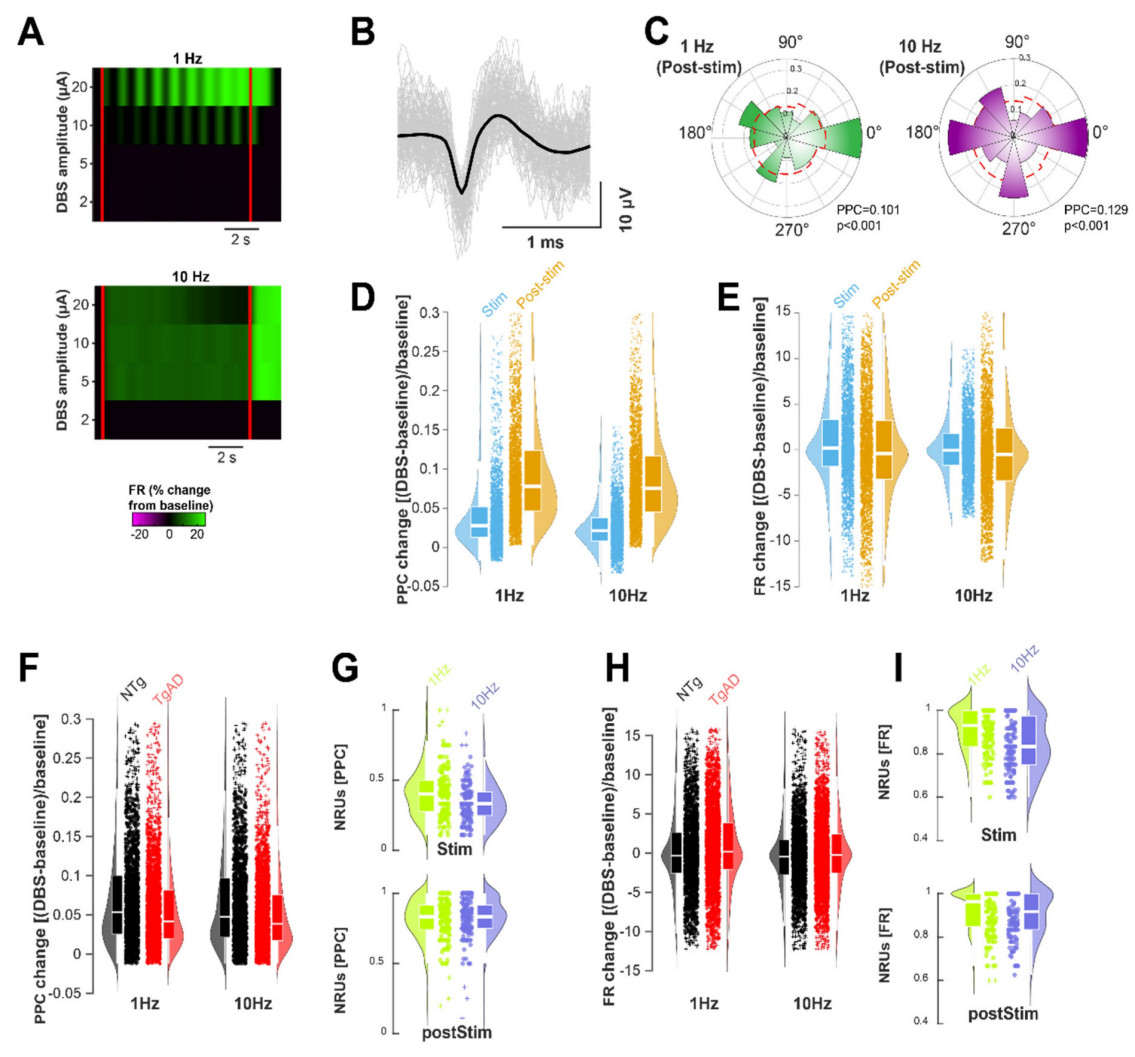
*DBS-elicited post-stimulation entrainment in TgAD rats with greater neuronal recruitment by 1 Hz than by 10 Hz DBS.* (A) Representative spiking unit showing changes in its firing rate (FR) following DBS stimulation (start and end indicated by red lines) at different amplitudes (y-axis) and different frequencies (top panel: 1 Hz, bottom panel: 10 Hz). (B) Spiking unit characteristics: average waveform (black) with individual waveforms (gray) of a putative hippocampal neuron during recording. (C) Normalized polar-plot histogram of spike phases for two representative units following DBS (colored bins) compared to baseline (no stimulation, red-dotted line) indicating an increase in entrainment, i.e. with spike phases aligning to the stimulation. (D) Change in Pairwise Phase Consistency (PPC, calculated from all spike phases) during stimulation (light blue) and following stimulation (orange) at different DBS frequencies. (E) Change in Firing Rate (FR) during DBS (light blue) and after DBS (orange) at different frequencies. (F) Change in PPC relative to pre-stimulus baseline of spiking units in NTg (black) and TgAD (red) rats at 1 Hz (left) and 10 Hz (right): mean change at 1 Hz: NTg = +9.9%±14.8% vs. TgAD = +7.1%±11.2%, p < 0.001; mean change at 10 Hz: NTg = +7.8%±14.2% vs. TgAD = +6.2%±10.5%, p < 0.001. (G) Number of responding units (NRU), expressed as a percentage of the total number of recorded units, showing significant PPC changes during (top) and after (bottom) DBS, in response to different DBS frequencies (1 Hz, green vs. 10 Hz, purple). The number of neurons entrained during 10 Hz DBS was 30.3±16.4%; while the number of neurons entrained post-10 Hz DBS was significantly higher, namely, 81.6±15.7% p < 0.001. (H) Change in spiking units' FR relative to pre-stimulus baseline in NTg (black) and TgAD (red) in response to 1 Hz DBS (left) and 10 Hz DBS (right). (I) Number of spiking units showing significant FR changes during (top) and post (bottom) DBS, in response to different DBS frequencies (1 Hz, lime vs. 10 Hz, lavender). Pairwise test for significance performed using Tukey-Kramer post-hoc test.

We analyzed spiking activity in two temporal windows: during DBS and post-DBS (Figure 5A), excluding frequencies >10 Hz due to limited temporal resolution. We quantified firing rate (FR) and pairwise phase consistency (PPC) in isolated units across DBS frequency and amplitude (Figure 5A-I). We assessed changes from baseline and defined the number of responding units (NRU) as those showing significant changes in FR or PPC during and after DBS (Figure 5G,I). GLME analysis showed that DBS frequency (but not amplitude) significantly affected NRU (p < 0.001 for both PPC and FR; Figure 5G,I) and PPC entrainment (p < 0.001; Figure 5D), while the effect on FR was marginal (p=0.064; Figure 5E). FR and PPC distributions were narrower at 10 Hz than at 1 Hz, with significantly reduced standard deviations: PPC from 0.046 to 0.030, FR from 5.22 to 3.30 (both p < 0.001, Levene's test). These results suggest that *increasing DBS frequency from 1 to 10 Hz leads to a more consistent neural response*, as reflected by reduced variability in PPC and FR across neurons.

DBS frequency exerted genotype-specific effects on both PPC and FR responses (p=0.0449 and p=0.0022, respectively; Figure 5F,H), indicating selective sensitivity of AD-affected neurons. A significant interaction between DBS frequency and time window (during vs. post-DBS) was observed for NRU in both FR and PPC (p = 0.005 and p = 0.002, respectively; Figure 5D,E), irrespective of genotype. Fewer neurons were entrained at 10 Hz than at 1 Hz in both genotypes; PPC responders dropped from 39.8% (CI: 36.9 to 42.6%) at 1 Hz to 30.3% at 10 Hz (Figure 5E; p < 0.001). Surprisingly, the NRU increased further during the post-stimulation period: for example, the NRU increased from 39.8% during 1 Hz DBS to 81.0% post 1 Hz DBS (CI: 78.9% to 83.1%, p < 0.001; Figure 4E), indicating a *strong post-DBS entrainment*. Overall, HIP TgAD neurons showed reduced level of entrainment response compared to NTg: at 1 Hz, PPC increased by +9.9% (CI: 9.2% to 10.5%) in NTg vs. +7.1% (CI:6.7% to 7.6%) in TgAD; Figure 4D, p < 0.001). FR increases were large and variable in both genotypes, for instance +73.5% (CI: -20% to 167%) for NTg and +93.7% (CI: 26.9% to 160.6%) for TgAD at 1 Hz; Figure 4F, p=0.987.

To examine the effects of DBS frequencies >10 Hz on neuronal firing and the potential confounding influence of anesthesia, we quantified post-DBS firing rates (FR) across all frequencies (1-130 Hz) and compared anesthetic conditions (Figure S4A-E). Although all FR responses were variable across the different DBS conditions, post-DBS FR tended to be higher under propofol than under isoflurane, with no significant modulation by DBS frequency or amplitude. Paralleling the effects observed in power and PAC, genotype-dependent differences in post-DBS firing rate responses were also significantly modulated by anesthetic (interaction effect, p = 0.009).

## Discussion

### Advanced AD pathology leads to power and modulation index attenuation

Electrophysiological recordings revealed marked differences between TgAD rats and age-matched non-transgenic animals, particularly in local field potentials (LFP) power and cross-frequency coupling (modulation index, MI). These differences reflect neurodegeneration and disrupted hippocampal network synchronization characteristic of advanced AD 29,32. Changes in LFP power were widespread and pronounced across all frequency bands, while deficits in modulation (MI) were concentrated in theta and delta-low gamma pairs. This selective disruption is consistent with the functional role of theta-gamma PAC in coordinating hippocampal information processing, especially during memory encoding and retrieval 33,34. These effects were not attributable to anesthesia depth, as physiological parameters were similar in both genotypes, confirming that the differences were specific to synaptic dysfunction and connectivity loss. The decline in power and phase-amplitude coupling (PAC) in the 16-month-old TgAD rats reflects disease progression compared to our prior observations of PAC attenuation in 9-month-old rats 32.

### Neuromodulation with DBS

We investigated the effects of DBS on the AD pathology affected brain by targeting the hippocampal (HIP) network, severely affected in AD. Standard DBS protocols often yield inconsistent outcomes, likely due to the diversity of neuronal circuitry and variability in disease progression, emphasizing the need for more sensitive assessments and personalized treatments 35. To date, only a few studies have systematically investigated the effects of DBS on brain networks, and they have largely neglected low frequencies (<40 Hz), despite their potential to match the endogenous firing patterns of hippocampal neurons, which typically fire at an average of about 2 Hz 14,36. In contrast, high-frequency DBS, commonly used to restore excitatory/inhibitory balance in neurodegenerative conditions 8,37, preferentially recruits inhibitory interneurons 38. While potentially beneficial, this approach may also produce functional lesions 39,40 and suppress low-firing neuronal activity by limiting repolarization time 41. As a result, high-frequency DBS can impair theta coupling that is essential for memory and spatial navigation, and is already compromised in AD 42. To better understand the effects of DBS on the local HIP network affected by AD pathology, we systematically explored a range of DBS parameters, using charge densities (amplitudes) consistent with previous reports 43,44 and selecting frequencies spanning the entire oscillatory range of HIP neurons. We assessed how DBS modulates neuronal power, modulation index (MI), pairwise phase consistency (PPC), and firing rate (FR) in the context of AD pathology and modeled the data to provide a predictive framework for estimating stimulation outcomes.

### DBS parameters influence the local HIP network

DBS is believed to produce an “information lesion” by overriding intrinsic neuronal activity and suppressing low-frequency oscillations within the targeted network, a mechanism referred to as synaptic filtering 45. This disruption of endogenous rhythms is expected to affect PAC, and manifests as changes in the modulation index (MI). In our data, DBS consistently induced PAC hyper-coupling (markedly elevated MI values), regardless of stimulation parameters. By imposing a stable oscillatory drive, DBS entrains local circuits and promotes excessive cross-frequency coupling (PAC hyper-coupling). PAC and power showed distinct sensitivities to DBS frequency: while PAC increased nonlinearly with frequency, power rose roughly linearly. This divergence suggests that power may be a more reliable marker of immediate network recruitment 46 in response to DBS, whereas PAC, though less suited to capturing graded, real-time responses, may offer a valuable readout of long-term circuit reorganization or dysfunction in unstimulated conditions 47.

At the single-neuron level, entrainment (measured by pairwise phase consistency, PPC) and firing rate (FR) were more responsive to changes in DBS frequency than DBS amplitude (especially so for PPC), confirming a strong entrainment even at the individual neuron level. We note that the weak dependency on amplitude may simply result from the spike-detection (AP) band's high-frequency nature, which limits its detection radius and causes depolarization of nearby neurons even at the lowest charge densities. Conversely, the effective detection radius for local field potentials (LFPs) may be larger than the stimulation radius, making LFP-based parameters like power and MI dependent on amplitude, as our findings suggest. Notably, changes in entrainment (PPC) were more pronounced during the post-DBS period in the 1-10Hz range, which may account for the significant increases in PAC observed during this window. This suggests that the duration of stimulation-free intervals (duty cycle) could be critical for optimizing DBS efficacy. Despite the complexity of the relationship between DBS parameters and network responses, key features emerged: while frequency band selectivity was limited to power responses, both MI and power responses increased with increases in DBS amplitude and frequency, highlighting their role in guiding parameter selection and maximizing DBS efficacy.

### DBS and AD pathology

Overall, we observed attenuated responses to DBS in both LFP power and MI in the HIP of TgAD rats, when compared to NTg littermates. The attenuated hippocampal power responses of TgAD to DBS align well with the notion of reduced HIP excitability 48, loss of synaptic activity 49, and frank neuronal loss observed in AD and in the TgAD rat model used here. PAC responses in TgAD were attenuated primarily at lower stimulation frequencies and amplitudes, suggesting altered thresholds for neuronal and network recruitment in the diseased brain, and underscoring the importance of tailoring DBS parameters to disease stage. Spiking unit analysis revealed that genotype differences depended more on DBS frequency than on amplitude. Despite exhibiting similar changes in FR, TgAD neurons were less entrained than NTg neurons in the low-frequency range (1-10Hz DBS). Altogether, our findings highlight the therapeutic potential of DBS in AD, and emphasize that DBS parameter optimization can, at least transiently, reverse the AD pathology-induced attenuation of neuronal activity. Future longitudinal studies using electrophysiological biomarkers and adaptable closed-loop protocols will be essential to assess the long-term efficacy of DBS in advanced AD.

### Therapeutic potential: retain and re-train

While prevention and early detection would be ideal, in the absence of a cure and given the slow progression of the disease, addressing neuronal dysfunction in symptomatic and advanced-stage AD will remain highly impactful for years to come. In advanced stage AD, DBS has been shown to slow disease progression 50,51. We propose that DBS may not simply stimulate surviving neurons in AD-affected regions to promote their retention under the “use it or lose it” paradigm, but that DBS may also serve to re-train the partially disconnected network. Some of the network's neurons, while partially deprived of inputs and not fully active, remain potentially responsive to external stimulation. We hypothesized that applying DBS within the functional frequency range (i.e. physiological oscillation bands within which neurons operate 52) allows for retaining and strengthening of their existing connections, ultimately promoting functional network reorganization and efficient information processing. Although baseline PAC and power were reduced and DBS responses attenuated, pointing to a shift in thresholds for neuronal recruitment by AD pathology, we showed that frequency-selective DBS could transiently normalize these deficits in the advanced stage of AD progression, underscoring the need for disease stage-specific optimization of neuromodulatory protocols (DBS parameters). While the mechanisms through which DBS produces neuromodulation are still incompletely understood 53, our study shows that precise DBS parametrization can enhance local network responsiveness and help re-establish more physiological activity patterns in hippocampal circuits disrupted by AD pathology. These findings may thus inform future translational efforts aimed at optimizing neuromodulation strategies for improving hippocampal network function in clinical AD.

## Supplementary Material

Supplementary methods and figures.

## Abbreviations

AD: Alzheimer's Disease
ANOVA: ANalysis Of VAriance
AP: Antero-Posterior
AP_band_: Action-Potential (band)
bpm: Beats per Minute
BR: Breathing Rate
brpm: Breaths per Minute
CSD: Current Source Density
DBS: Deep Brain Stimulation
DV: Dorso-Ventral
FR: Firing Rate
GLME: Generalized Linear Mixed Effects
HIP: Hippocampus
HR: Heart Rate
Hz: Hertz
LFP: Local Field Potential
LMA: Linear Multi-Array
MI: Modulation Index
ML: Medio-Lateral
MUA: Multi-Unit Activity
NRU: Number of Responding Units
NTg: Non-Transgenic
PAC: Phase-Amplitude Coupling
pO2: Partial (pressure) of Oxygen
PPC: Pairwise Phase Consistency
SD: Standard Deviation
TgAD: Transgenic Alzheimer's disease rat model (F344-AD)
