# Supplementary material for: Deep brain stimulation-induced normalization of hippocampal synchrony in a transgenic rat model of Alzheimer's disease
Source: Theranostics. 2025 Sep 22;15(18):10064–75. doi: 10.7150/thno.110292 (PMC12486142; doi:10.7150/thno.110292)
Supplement: Supplementary file 1 — Supplementary methods and figures. [file thnov15p10064s1.pdf]

# SUPPLEMENTARY MATERIAL

## Extended methods

**Animal Care and Surgical Procedure:**

TgF344-AD rats were utilized for this study as a model for Alzheimer's disease. TgF344-AD rats overexpress human APPswe and PS1ΔE9 under the control of the mouse prion promoter and are outbred on a Fischer 344 rat background. All animal procedures were conducted following the guidelines approved by the Animal Care Committee of the Sunnybrook Research Institute and in compliance with the ethical standards set forth by the Canadian Council on Animal Care. The rats were bred in-house and maintained on a 12-hour light-dark cycle with ad libitum access to chow and water. Sex-balanced and age-matched TgF344-AD (TgAD) and non-transgenic (NTg) littermate rats were used in this study. The rats' age was 15.8 ± 0.1 months at the time of the study and their mean body weight was 322.1 ± 40.0 grams (females), and 533.2 ± 47.6 grams (males). Rats' physiology was monitored during anesthesia using the MouseOx system equipped with a rat foot sensor to report changes in oxygen saturation, breathing, and heart rate. The rats were anesthetized with isoflurane (5% induction, 2-2.5% maintenance) and the center of the craniotomy was marked at AP −3.5 mm from bregma and ML −2.5 mm. The anesthetized rats underwent a craniotomy procedure (3 mm in circumference) followed by a durotomy, performed as previously described [1] over the left hemisphere, to provide access for the electrophysiological recordings and DBS.

**Electrophysiology:**

One double-shank linear multielectrode array (2×6 LMA electrode, Microprobes) was inserted into the left hippocampus (HIP), oriented to span the dorsoventral axis and with the shanks aligned with the hippocampal anatomy (approximately 30° counterclockwise in the transverse plane, relative to the bregma-lambda axis). The electrode tip was inserted to a depth of 6.0 mm from the brain surface (DV), thus placing the deepest electrode site at 4.5mm. The LMA electrode sites were 25 μm in diameter and were spaced 500 μm along the dorsoventral axis (distance between sites) and 400 μm along the mediolateral axis (distance between shanks). The averaged LMA electrode site impedance was 152.3 ± 6.6 kΩ (measured

at 1 kHz). The electrode was connected to a switching headstage (Tucker and Davis Technologies, TDT), which allowed for very fast (<0.2 ms) switching between DBS stimulation and recording. The electrophysiological signals were acquired using a TDT 32-channel system.

**Anesthesia Transition and Deep Brain Stimulation (DBS) parameters:**

After the surgical procedure, to provide a stable state of light anesthesia, rats were transitioned from maintenance isoflurane (2-2.5%) to low isoflurane (1.25-1.5%) and propofol by intravenous injection via a tail vein catheter. The induction dose of propofol was 7.5 mg/kg, and maintenance was achieved at 40-50 mg/kg/hr. TDT subject interface was used to record and deliver stimulation. DBS was applied using bipolar mode through the top and bottom contact sites of each shank of the LMA electrode, thereby passing current throughout the entire hippocampus. Baseline recordings were performed under low isoflurane and propofol before DBS was delivered using a 0.05 ms square pulse at varying current amplitudes, presented in the following order: 2, 5, 10, and 20 μA, corresponding to estimated charge densities of 20, 50, 100, and 200 $\mu C/cm^2$ per pulse, respectively. These amplitudes were tested in combination with six stimulation frequencies: 1, 10, 20, 40, 100 and 130 Hz. Each DBS block, defined by a specific amplitude and frequency combination, lasted 10 seconds, followed by a 20-second recovery period (post-DBS) before the next amplitude was presented. A full DBS segment, consisting of all amplitude conditions at a given frequency, lasted 120 seconds and was followed by a 5-10 minute interval before switching to the next stimulation frequency. For the long DBS duration DBS was presented at 10 Hz/20 μA for a total of 900 seconds (15 min), followed by 20 min of no stimulation where the persistence of DBS effects was analyzed. For the 1-10 Hz propofol DBS recording a total of 19 and 17 rats were used per group (NTg and TgAD respectively) while for the low-isoflurane vs. propofol, 20-130Hz DBS frequencies and short (10 s) vs. long (900 s) DBS recordings 4 rats were used per group.

**Data Analysis and Presentation:**

Electrophysiological signals from the 2x6 LMA were acquired at 24.4 kHz and raw data was imported in MATLAB (MathWorks) using TDT offline analysis tools (*TDTbin2mat* function) and routine analysis was performed following adaptation from previous work [1–3]. Raw electrophysiological traces were filtered

using 2nd-order low-pass and high-pass digital filters with cutoffs at 300 Hz and 750 Hz, respectively. This filtering process separated the electrophysiological signals into two frequency bands: the low-frequency band (local field potentials, LFP: 0.3–300 Hz) and the high-frequency band (AP band: 0.75–3 kHz). Following the separation of LFP, power was computed in the 1-120 Hz range in the post-stimulation period (2 sec) using MATLAB's built-in *spectrogram* function, which applies the short-time Fourier transform with a Hamming window. Cross-frequency coupling was calculated from the LFP band over the 1-120 Hz range in the same post-DBS window (2 sec) and reported as the “Modulation Index” (MI), which quantifies the strength of PAC by measuring the extent of modulation of the high-frequency amplitude (30-120 Hz oscillations) by the phase of low-frequency (1-20Hz) oscillations [4]. The two seconds window immediately following the end of DBS, used to calculate power and modulation index, provides sufficient resolution for the lowest frequency analyzed (1 Hz) before stimulation onset, to establish baseline conditions, and after stimulation to capture the network's response to DBS. The AP band was analyzed in Kilosort 3 [5] for spike detection and clustering. For Kilosort analysis, registration was disabled (ops.nblocks = 0), and threshold parameters were set for spike detection as follows: ops.spkTh = -5 and ops.Th = [8 5]. All other parameters were kept at their default settings. The spiking data of the sorted clusters were further analyzed for changes in the firing rate (estimated from the inter-spike intervals) and for changes in the pairwise-phase consistency (PPC) during and after stimulation [6]. For the PPC calculation, the phase of the stimulation was derived by fitting a continuous sinusoidal phase to the DBS pulse train based on pulse peaks. The phases for each spike relative to this stimulation phase were then extracted using Hilbert's transform. The PPC was then computed as the cosine of the absolute angular distance between all spike phase pairs. The square root of the absolute PPC value ($\sqrt{|PPC|}$) was then used to estimate the conventional phase-locking value (PLV), where 0 indicates no entrainment and 1 signifies complete entrainment. The $\sqrt{|PPC|}$ (referred to as 'PPC' in the following sections), equivalent to the population statistic of the PLV, is computed for all putative neurons (units) within the two-second window at baseline (before DBS onset), during DBS, and post-DBS. The number of responding units (NRU), defined as units exhibiting significant changes in PPC or firing rate (FR) during and after stimulation, was determined by generating a null distribution through random shuffling of all FR/PPC values of unit. These shuffled values were then assigned to three blocks (pseudo-baseline, pseudo-stimulation, and pseudo-post-stimulation) repeated 1E4 times. For each iteration, the median

values were calculated, and the change (delta) was computed by comparing the pseudo-stimulation and pseudo-post-stimulation to the pseudo-baseline. This resulted in 10,000 pseudo-change values, forming a null distribution. The observed changes in FR and PPC were then compared to this null distribution, and a two-sided p-value was then calculated to assess significance. Each unit was then classified as responding if the p-value was less than 0.01, and the NRU was then calculated as the ratio of responding to total units. Changes in the parameters (power, MI) were calculated and presented as: ∆Param = Param[DBS] - Param[baseline], while changes in PPC and FR were presented as ΔParam = (Param[DBS] – Param[baseline]) / Param[baseline]. Raincloud plots in Figure 1 and 5 were prepared using the online statistical visualization function package *DataViz* [7], while raincloud plots presented in Figure 2 were prepared using *R* library ggdist (stat_halfeye).

**Statistical and power analyses:**

Linear mixed-effects models (LMMs; ImerMod objects from the *Ime4* package in *R*) were used to examine the relationship between the response variables and fixed effects, including genotype, sex, anesthetic, and DBS parameters. These models accounted for hierarchical data structure, interaction effects, and variability at the individual or channel level (random effects). Response variables were transformed as needed to meet the assumption of normally distributed residuals, verified using diagnostic tools from the *DHARMa* package in *R*. Model selection was conducted using a genetic algorithm (*glmulti* package), and the top-ranked models within 3 Akaike Information Criterion (AIC [8]) units of the best model were averaged to generate predictions.

For the physiological parameters BR, HR, and pO2, the following model was employed:

*'[HR/BR/pO2] ~ Genotype * Sex * AnesthesiaType + (1|Subject)'*

GLME models were used to examine the cross-frequency coupling and the power spectrum at baseline using the following formula:

*'[MI/P] ~ Genotype * Sex * Frequency_band + (1|Subject:Channel)'*

and during DBS:

*‘deltaMI ~ Anesthetic + HIP + Genotype + Ph2Amp + DBS_amp + DBS_freq + Anesthetic:HIP + Anesthetic:Genotype + Anesthetic:DBS_amp + Anesthetic:DBS_freq + HIP:Ph2Amp + HIP:DBS_freq + Genotype:DBS_amp + Genotype:DBS_freq + Ph2Amp:DBS_amp + Ph2Amp:DBS_freq + DBS_amps:DBS_freq + (1|Subject:Channel)’*

*‘deltaP ~ Anesthetic + HIP + Genotype + Ph2Amp + DBS_amp + DBS_freq + Anesthetic:HIP + Anesthetic:Genotype + Anesthetic:DBS_amp + Anesthetic:DBS_freq + HIP:Ph2Amp + HIP:DBS_amp + Genotype:Ph2Amp + Genotype:DBS_amp + Ph2Amp:DBS_amp + Ph2Amp:DBS_freq + DBS_amp:DBS_freq + (1|Subject:Channel)’*

The GLME models used to evaluate entrainment (PPC, change from baseline and number of responding units, NRU) were specified as follows:

*‘deltaPPC/NRU ~ Genotype * DBS_Frequency * DBS_Window + (1|Subject:Cluster)'*

While the firing rate modulation was modeled by:

*‘deltaFR ~ Anesthetic + Genotype + DBS_amp + DBS_freq + Anesthetic:Genotype + Anesthetic:DBS_amp + Anesthetic:DBS_freq + (1|Subject:Cluster)'*

Where ‘DBS_Window’ refers to the window of analysis (during and post-stimulation). Pairwise comparison was performed following a linear n-way analysis of variance (ANOVA, *‘anovan’* function in MATLAB), using multiple comparison analysis with Tukey-Kramer correction for family-wise error rate (*‘multcompare’* function in MATLAB and emmeans library in *R*). To estimate the sensitivity of our design, we conducted a post hoc power analysis using the pwr package in *R* (pwr.t2n.test) with the observed effect sizes (Cohen’s *d*) from key group comparisons. For the baseline MI, effect sizes across the 16 pairwise comparisons in Figure 2 ranged from $d$=0.003 to 0.80. At $\alpha$=0.05 (two-sided), the corresponding power ranged from 0.05 to 0.64, with two comparisons (D-LG and D-V) reaching power >0.6. In contrast, the baseline power comparisons showed large effect sizes across all 12 conditions ($d$=1.70 to 3.25), yielding power estimates consistently above 0.99.

# Extended Results

**Systemic state**

The rats were transitioned from isoflurane to propofol intravenous infusion, which was adjusted to maintain stable physiological parameters across subjects (mean heart rate, HR = 328.6 bpm, CI=311.4-345.7 for NTg; HR = 324.8 bpm, CI=310.7-338.8 for TgAD and mean breath rate, BR = 57.2 brpm, CI=54.3-60.1 for NTg and BR = 57.4 brpm, CI=53.7-61.1, Figure 1G-I). The switch to propofol aimed to produce a light state of anesthesia that preserves functional connectivity patterns similar to the awake state [9] during DBS and electrophysiological recordings. While we found a significant interaction between sex and type of anesthesia (isoflurane vs propofol) on HR (p= 0.015) and BR (p=0.04), no differences were found for the effect of the interaction between genotype (TgAD vs. NTg littermates) and type of anesthesia on HR or BR (p=0.209 and p=0.534, respectively). No differences in heart rate, breathing rate, or partial $O_2$ saturation were observed between genotypes during propofol following multiple comparisons (p= 0.97, p= 0.99, and p= 0.94 respectively).

# SUPPLEMENTAL FIGURES

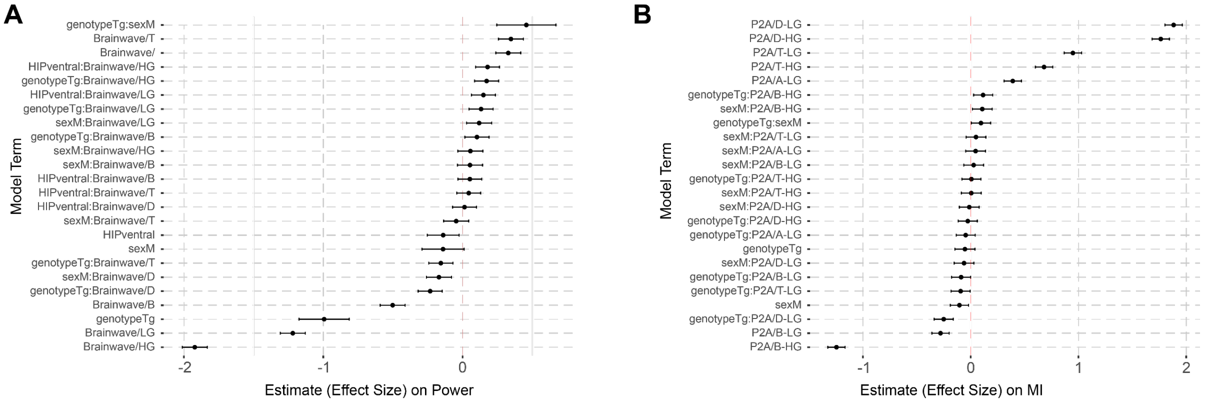


**Figure S1. *Effect estimates on LFP at baseline*. (A-B)** Forest plot showing the effect sizes (model estimates ± 95% CI) for each predictor in the linear mixed-effects model used to analyze baseline LFP power (A) and MI (modulation, B) in TgF344AD rats. Key contributors include genotype, sex, brainwave (aka oscillation band), and P2A (phase-amplitude comodulation) interactions.

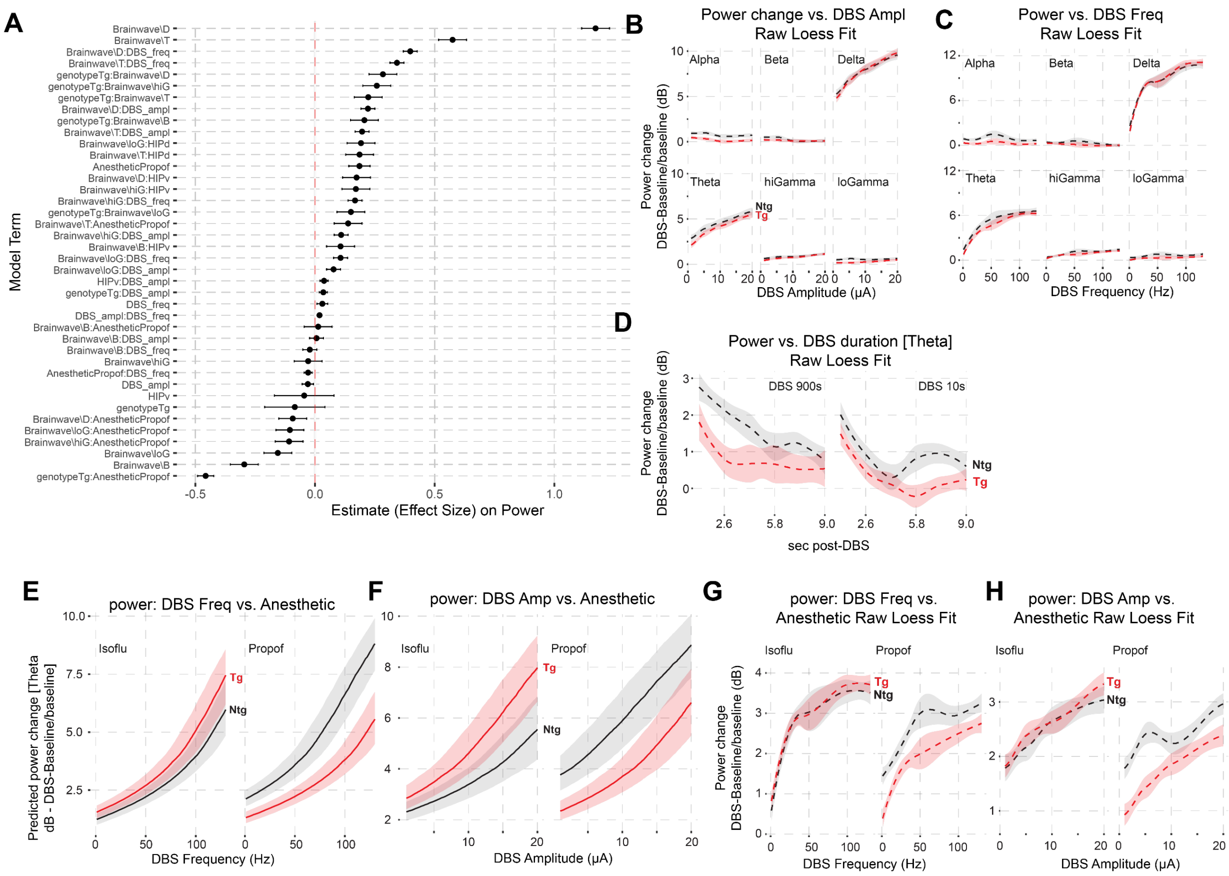


**Figure S2.** ***Modeling and raw data analysis of DBS-induced hippocampal power responses across genotypes, parameters, and anesthetics.*** **(A)** Forest plot showing the effect sizes (model estimates ± 95% CI) for each predictor in the linear mixed-effects model used to analyze post-DBS changes in power. Key contributors include DBS frequency, amplitude, brainwave (aka oscillation band), and genotype interactions. **(B)** Raw data smoothed using LOESS regression illustrating the relationship between DBS amplitude and post-stimulation power across oscillation bands (alpha to high gamma) in TgAD (red) and NTg (black) rats. These plots correspond to modeled results shown in Figure 3E. **(C)** LOESS fits showing the impact of DBS frequency on power responses by genotype and band, corresponding to modeled data in Figure 3D. **(D)** LOESS plots illustrating post-DBS power decay over time (0–10 s) in the theta band after short (10 s) and long (900 s) stimulation trains, comparing NTg and TgAD rats. The plot refers to the persistence of DBS effects on power shown in Figure 3F. **(E-F)** Model-based predictions of theta-band power increases as a function of DBS frequency (E) and amplitude (F), separated by anesthetic (isoflurane: left, propofol: right). Under isoflurane, DBS-induced power increases are generally larger in TgAD (red) than in NTg (black), whereas this pattern is reversed under propofol, where NTg animals exhibit stronger power enhancements. **(G-H)** LOESS fits of raw data underlying the models shown in (E-F), illustrating anesthesia-dependent trends in power response to DBS frequency (G) and amplitude (H). Across both anesthetics, TgAD rats show attenuated responses compared to NTg controls.

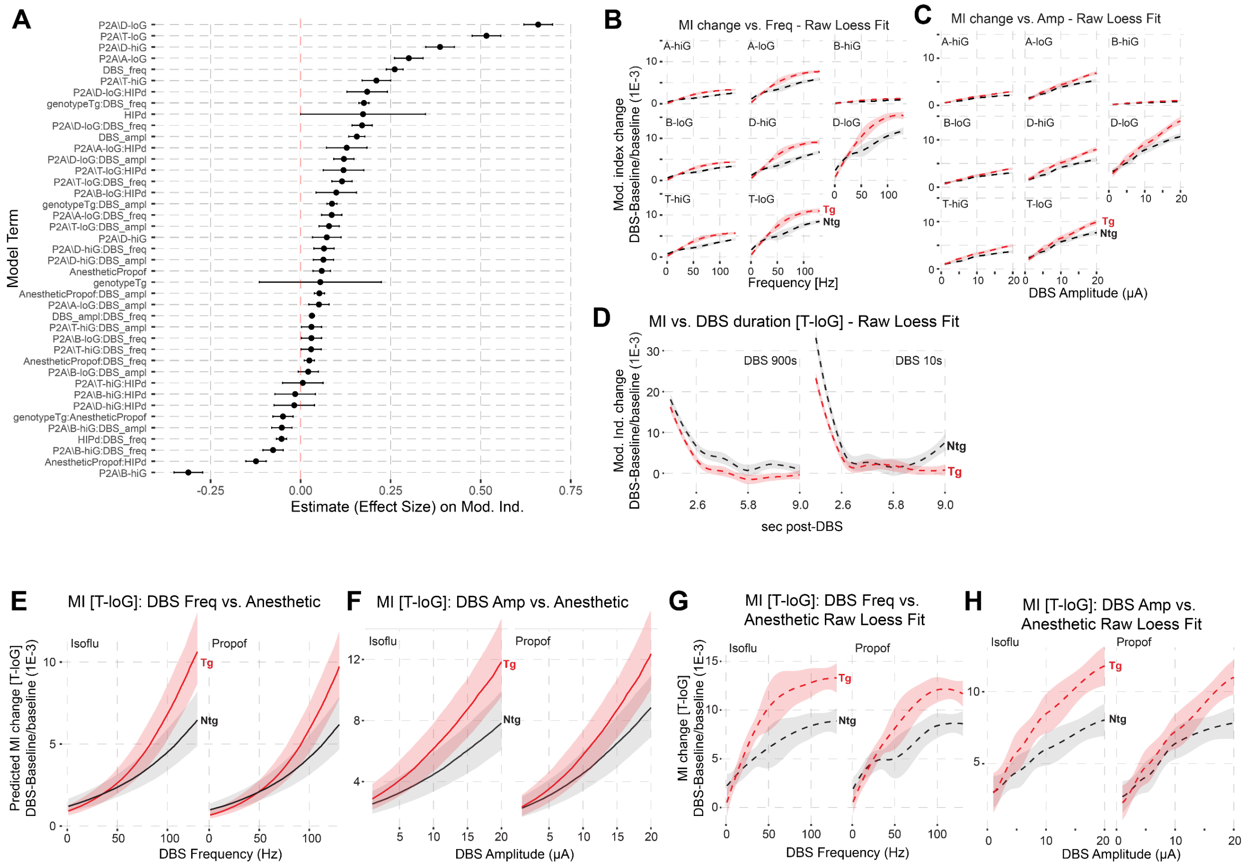


**Figure S3:** ***Modeling and raw data analysis of DBS-induced hippocampal PAC (modulation index) changes across genotypes, parameters, and anesthetics.*** **(A)** Forest plot showing the effect sizes (model estimates ± 95% CI) for each predictor in the linear mixed-effects model used to analyze post-DBS changes in MI. Key contributors include DBS frequency, amplitude, brainwave (aka oscillation band), and genotype interactions. **(B)** Raw data smoothed using LOESS regression illustrating the relationship between DBS amplitude and post-stimulation MI across combination of PAC oscillation bands (delta (D), theta (T), alpha (A), beta (B) vs. Low (LoG) and High (HiG) gamma) in TgAD (red) and NTg (black) rats. These plots correspond to modeled results shown in Figure 4C. **(C)** LOESS fits showing the impact of DBS frequency on MI by genotype and PAC, supporting modeled data in Figure 4C. **(D)** LOESS plots illustrating post-DBS MI decay over time (0–10 s) in the theta-low gamma PAC following short (10 s) and long (900 s) DBS, comparing NTg and TgAD rats. The plot refers to the persistence of DBS effects on MI shown in Figure 4E. **(E-F)** Model-based predictions of MI (PAC = theta-low gamma) increases as a function of DBS frequency (E) and amplitude (F), separated by anesthetic (isoflurane: left, propofol: right). Regardless of anesthetic used, DBS-induced increase in MI were generally larger in TgAD (red) than in NTg (black). **(G-H)** LOESS fits of raw data underlying the models shown in E–F, illustrating anesthesia-dependent trends in MI response to DBS frequency (G) and amplitude (H) for theta-low gamma PAC. Across both anesthetics, TgAD rats show enhanced MI responses compared to NTg controls.

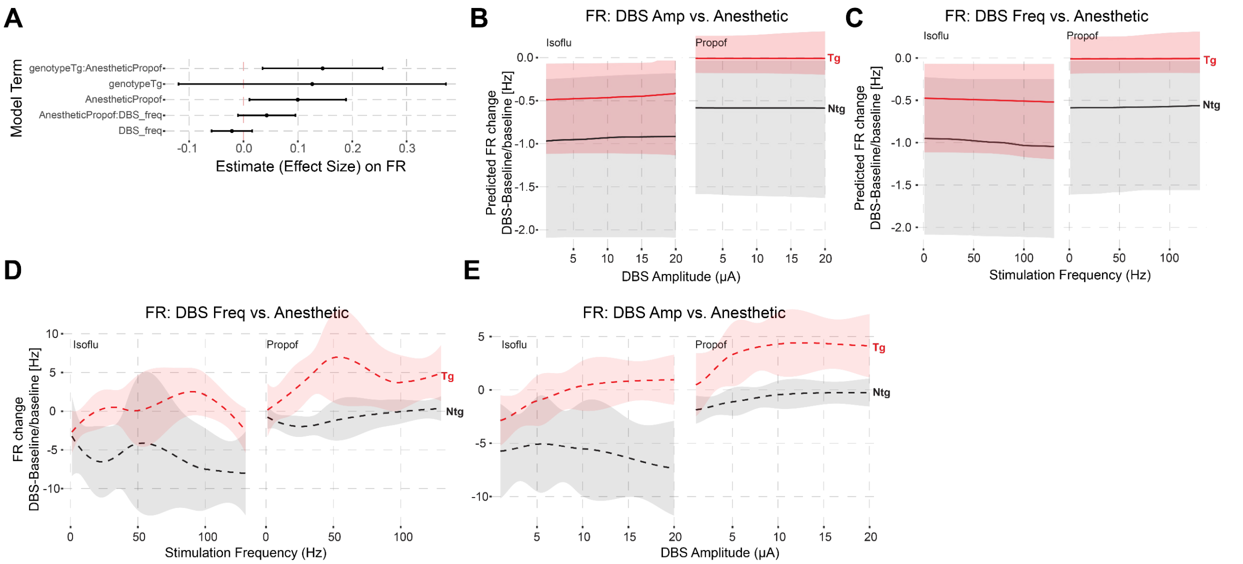


**Figure S4.** ***DBS-induced Changes in Firing Rate (FR) in TgAD Rat Model*. (A)** Forest plot showing the effect sizes (model estimates ± 95% CI) for each predictor in the linear mixed-effects model used to analyze post-DBS changes in firing rate. **(B-C)** Model-based predictions of changes in firing rate ((DBS-Baseline)/Baseline) as a function of DBS amplitude (B) and frequency (C), separated by anesthetic (isoflurane: left, propofol: right). TgAD: red, NTg: black. **(D-E)** LOESS fits of the raw data illustrating the relationship between DBS frequency (D) and amplitude (E) and the post-DBS change in firing rate. These plots show the underlying raw data trends that support the models presented in panels (B) and (C).

# REFERENCES


1. Bazzigaluppi P, Beckett TL, Koletar MM, et al. Early-stage attenuation of phase-amplitude coupling in the hippocampus and medial prefrontal cortex in a transgenic rat model of Alzheimer's disease. J Neurochem. 2018; 144: 669–79.

2. Canolty RT, Knight RT. The functional role of cross-frequency coupling. Trends Cogn Sci. 2010; 14: 506–15.

3. Tort ABL, Komorowski RW, Manns JR, Kopell NJ, Eichenbaum H. Theta-gamma coupling increases during the learning of item-context associations. Proc Natl Acad Sci U S A. 2009; 106: 20942–7.

4. Canolty RT, Edwards E, Dalal SS, et al. High gamma power is phase-locked to theta oscillations in human neocortex. Science. 2006; 313: 1626–8.

5. Pachitariu M, Sridhar S, Stringer C. Solving the spike sorting problem with Kilosort. bioRxiv. 2023 Available at: https://www.biorxiv.org/content/10.1101/2023.01.07.523036v1

6. Vinck M, van Wingerden M, Womelsdorf T, Fries P, Pennartz CMA. The pairwise phase consistency: a bias-free measure of rhythmic neuronal synchronization. Neuroimage. 2010; 51: 112–22.

7. Karvelis P. povilaskarvelis/DataViz: v3.2.4. 2024. Available at: https://zenodo.org/records/12749045

8. Burnham KP, Anderson DR. Model Selection and Multimodel Inference. Springer New York; 2002.

9. Paasonen J, Stenroos P, Salo RA, Kiviniemi V, Gröhn O. Functional connectivity under six anesthesia protocols and the awake condition in rat brain. Neuroimage. 2018; 172: 9–20.
